# Targeting tubular NAT10 ameliorates the renal Tubulointerstitial Fibrosis in Obstructive and Folic Acid-Induced Nephropathy

**DOI:** 10.7150/ijbs.135844

**Published:** 2026-09-02

**Authors:** Qin Kang, Lin Yin, Chenkai Wu, Wende Yan, Sai Peng, Jinfang Lu, Dongshan Zhang

**Affiliations:** 1 Department of Critical Medicine, the Second Xiangya Hospital, Central South University, Changsha, Hunan 410011, China.; 2 Department of Biology and Medicine, Central South University of Forestry and Technology, Changsha, Hunan 410004, China.; 3 Department of Emergency Medicine and Difficult Diseases Institute, Central South University, Changsha, Hunan, China.; 4 The Second Affiliated Hospital of Nanchang University, Nanchang 330006, Jiangxi, China.; 5 Hunan Aerospace Hospital, Changsha 410205, Hunan, China.; 6 Department of Nephrology, The First Affiliated Hospital of University of South China, Hengyang 421001, Hunan Province, China.; 7 Department of Ophthalmology, The Second Xiangya Hospital, Central South University, Changsha 410011, China.

**Keywords:** chronic kidney disease, NAT10, renal fibrosis

## Abstract

Renal tubulointerstitial fibrosis (TIF) represents a common pathological hallmark during the progression of chronic kidney disease (CKD). NAT10, an RNA acetyltransferase, catalyzes N4-acetylcytidine modification of mRNA and has been associated with multiple pathological events, including bladder cancer progression, tumor metastasis, and cardiac remodeling. Nevertheless, NAT10's precise function and underlying molecular mechanisms in TIF remain inadequately elucidated. In this investigation, transforming growth factor-β1 (TGF-β1) was found to promote NAT10 expression in BUMPT cells via p53 upregulation. Functionally, NAT10 was demonstrated to regulate FN, Collagen I, and Collagen III expression triggered by TGF-β1 stimulation. Mechanistically, NAT10 enhanced Spred2 expression by promoting acetylation-dependent mRNA stability, which subsequently activated the JNK and ERK/TGF-β signaling pathways. Furthermore, a renal tubular epithelial cell-specific NAT10 knockout mouse model was established, and NAT10 deficiency's influence on TIF progression was examined using unilateral ureteral obstruction (UUO)- and folic acid (FA)-induced renal fibrosis models. The findings indicated that tubular epithelial-specific NAT10 deletion markedly alleviated UUO- and FA-induced renal TIF via inhibiting Spred2/JNK and ERK/TGF-β signaling. Finally, 5-Fluorouridine, a potent ribozyme self-cleavage inhibitor, was observed to attenuate UUO- and FA-induced renal TIF by targeting NAT10. Collectively, a previously unrecognized p53/NAT10/Spred2/JNK and ERK/TGF-β regulatory axis driving renal TIF is identified, and 5-Fluorouridine represents a viable therapeutic intervention for renal TIF.

## Introduction

Renal fibrosis serves as the ultimate shared pathway for advancing chronic kidney disease (CKD) and poses a major global health burden^1^. Tubulointerstitial fibrosis (TIF) triggered by renal tubular epithelial injury is acknowledged as a pivotal pathological mechanism in renal fibrosis progression. Although currently available therapeutic strategies (RAS blockage and SGLT2 inhibitors) can decelerate CKD progression^2^, these interventions exhibit limited efficacy in counteracting TIF. Therefore, further elucidation of the pathophysiological mechanisms underlying renal TIF and the identification of novel therapeutic approaches for preventing its progression remain urgently required.

RNA modifications, including N6-methyladenosine (m6A) and N4-acetylcytidine (ac4C), which constitute important components of the epitranscriptome, participate in the regulation of RNA metabolism and diverse cellular processes^3^. Previous studies have demonstrated that Mettl3 modulates target gene expression via m6A modification, thus facilitating the progression of acute kidney injury (AKI) and renal fibrosis^4^. Currently, ac4C is recognized as the only characterized acetylation modification identified in eukaryotic RNA, and NAT10 has been identified as the sole human enzyme possessing both acetyltransferase activity and RNA-binding capability^5^. Increasing evidence has linked NAT10 to the pathogenesis, progression, and prognosis of multiple disorders, including cancer and Hutchinson-Gilford progeria syndrome^6^-^8^. Furthermore, recent investigations have reported that the RNA acetyltransferase NAT10 facilitates myocardial fibrosis and pulmonary fibrosis^9^-^11^. However, the involvement of NAT10-mediated acetylation modification in renal fibrosis remains insufficiently characterized.

In this study, NAT10 was identified as a mediator of transforming growth factor-β1 (TGF-β1)-induced renal fibrosis. Mechanistically, Spred2 expression was elevated by NAT10 through acetylation-dependent stabilization of mRNA, which subsequently activated the JNK and ERK/TGF-β1 signaling pathways and facilitated TIF. A renal tubular epithelial cell-specific NAT10 knockout (T-NAT10-KO) mouse model was established, and T-NAT10-KO was observed to markedly alleviate UUO- and folic acid (FA)-induced renal fibrosis. Finally, the novel NAT10 inhibitor 5-Fluorouridine exerted comparable inhibitory effects and reduced UUO- and FA-induced renal fibrosis.

## Results

### NAT10 expression is upregulated by TGF-β1 in BUMPT cells and by UUO in mouse kidneys

Initially, it was examined whether NAT10 expression was induced during renal fibrosis. RT-qPCR analysis indicated that TGF-β1 induced NAT10 mRNA expression at 6 h, with progressive elevation at 12 h and peak levels achieved at 24 h (Figure 1A). These findings were further validated by immunoblot analysis showing a corresponding increase in NAT10 protein expression (Figure 1B and C). Immunofluorescence staining combined with semi-quantitative analysis demonstrated that NAT10 was predominantly localized in the nucleus and was substantially raised in BUMPT cells following 24 h TGF-β1 treatment (Figure 1D&E). To further validate the *in vitro* observations, a mouse UUO model was established. Kidney tissues were harvested at days 0, 3, and 7 following UUO surgery. H&E and MTS revealed progressive renal tubular injury and TIF, whereas immunohistochemistry staining demonstrated a gradual increase in NAT10 expression (Figure 1F-I). Subsequently, RT-qPCR and WB analyses demonstrated notable elevation of NAT10 mRNA and protein expression in UUO mouse kidneys (Figure 1J-L). Collectively, these observations demonstrate that NAT10 expression shows marked elevation in both TGF-β1-stimulated renal tubular epithelial cells and fibrotic kidneys from UUO mice, suggesting a potential association between NAT10 and renal fibrosis progression.

### P53 functions as a transcriptional activator to upregulate NAT10 expression

Previous studies have demonstrated that TGF-β can activate p53, NFκB, Smad3, and other signaling molecules. However, immunoblot analysis revealed that p53 knockdown resulted in the most pronounced reduction in NAT10 expression (Figure 2A-B). To determine the regulatory factor responsible for the elevation of NAT10 expression, bioinformatic analysis was conducted. The analysis revealed that potential p53-binding motifs exist in the promoter region of the mouse NAT10 gene (Figure 2C, predicted by http//molotool.autosome.org). ChIP assays further verified that p53 directly associates with two distinct sites in the NAT10 promoter region within mouse genomic DNA (Figure 2D). To obtain quantitative evidence for this interaction, we conducted ImageJ-based grayscale analysis on ChIP electrophoretic bands. The IgG group yielded no detectable target band, confirming the specificity of p53-mediated pulldown. By normalizing ChIP signals to the corresponding Input template of each sample group, we calculated the relative binding percentage, which confirmed the constitutive binding of p53 to the NAT10 promoter in both control and TGF-β1-treated tubular cells (Figure 2E-F). Dual-luciferase reporter assays demonstrated that p53 overexpression significantly enhanced luciferase activity in the wild-type NAT10 plasmid, whereas this increase was markedly attenuated in the mutant p53 plasmid with disrupted binding sites (Figure 2G). Subsequently, the expression patterns of p53 and NAT10 in BUMPT cells were examined following TGF-β1 stimulation. Immunoblot analysis revealed that TGF-β1 stimulated FN, Collagen I, Collagen III, p53, and NAT10 expression at the specified time points (Figure 2H-I). To further evaluate whether NAT10 expression is directly regulated by p53, loss- and gain-of-function experiments were executed. Notably, p53 knockdown markedly reduced TGF-β1-induced upregulation of NAT10 (Figure 2J-K). In contrast, p53 overexpression further increased NAT10 expression in response to TGF-β1 stimulation (Figure 2L-M). Collectively, these findings indicate that p53 serves as a transcription factor that directly binds to the NAT10 promoter and enhances its expression, thereby contributing to TGF-β1-induced fibrogenic responses.

### NAT10 facilitates TGF-β1-induced fibrotic marker expression in BUMPT cells

Although p53 enhances NAT10 expression in BUMPT cells after TGF-β1 treatment, the functional role of NAT10 in this process remains insufficiently defined. To clarify its involvement, NAT10-specific siRNA or an NAT10 overexpression plasmid was introduced into BUMPT cells, after which the expression of fibrosis-associated markers was evaluated. Immunoblot analysis demonstrated that TGF-β1 treatment (5 ng/mL) markedly elevated the protein levels of FN, Collagen I, and Collagen III. This elevation was substantially reduced following NAT10 knockdown, whereas NAT10 overexpression further augmented these effects (Figure 3A-D). Collectively, these observations suggest that NAT10 positively regulates TGF-β1-induced fibrotic responses in renal tubular epithelial cells.

### NAT10 enhances Spred2 mRNA stability via acetylation modification

NAT10, the only identified human enzyme with RNA acetyltransferase activity, has been reported to enhance the stability of downstream target transcripts through acetylation modification. To clarify the downstream mechanism through which NAT10 promotes renal fibrosis, bioinformatic analysis was conducted to screen for potential NAT10 target genes, leading to the identification of three candidate NAT10-binding regions within Spred2 mRNA (Figure 4A). To determine whether NAT10 influences Spred2 mRNA stability, BUMPT cells stably transfected with scramble-shRNA or NAT10-shRNA underwent treatment with actinomycin D (5 μg/mL), and Spred2 mRNA decay was subsequently evaluated at the indicated time points. The results demonstrated that NAT10 knockdown markedly enhanced Spred2 mRNA degradation, particularly at 4 h after treatment (Figure 4B), suggesting that NAT10 contributes to the stabilization of Spred2 mRNA. We performed ac4C-specific RIP-qPCR to directly detect ac4C modification on Spred2 mRNA (Figure 4C). We then performed ImageJ grayscale semi-quantification on the western blot bands from RIP products to quantify the binding enrichment level between NAT10 protein and Spred2 mRNA complex. The IgG group displayed nearly no specific signal, eliminating non-specific binding interference. After normalizing the signal of NAT10 immunoprecipitates to the Input loading control, we calculated the relative fold enrichment, which offered quantitative evidence for the physical interaction between NAT10 and Spred2 transcript (Figure 4D). Dual-luciferase reporter assays revealed that NAT10 overexpression markedly elevated luciferase activity in wild-type Spred2 plasmids, whereas no increase was observed in the mutant Spred2 plasmid containing disrupted binding sites (Figure 4E), indicating that NAT10 regulates Spred2 through sequence-specific interaction with its mRNA.

Additionally, the influence of NAT10 modulation on Spred2 expression was examined. RT-qPCR and WB analyses demonstrated that TGF-β1 triggered elevations in Spred2 mRNA and protein expression levels, which were diminished following NAT10 knockdown, while NAT10 overexpression additionally augmented this response (Figure 4F-K). Collectively, these observations demonstrate that NAT10 binds to Spred2 mRNA and enhances its stability, thus elevating Spred2 expression.

### Spred2 mediates the NAT10 effect on increasing fibrotic markers induced by TGF-β1 via activating the JNK and ERK/TGF-β axis

Although the preceding findings indicated that NAT10 increases Spred2 expression, the role and underlying mechanism of Spred2 in TGF-β1-induced renal fibrosis remain insufficiently defined. The findings revealed that Spred2 knockdown markedly diminished TGF-β1-induced elevation in FN, Collagen I, Collagen III, and TGF-β levels, along with JNK and ERK signaling activation (Figure 5A-D). To further determine whether Spred2 mediates the regulatory function of NAT10 during TGF-β1 stimulation, rescue experiments were conducted by co-transfecting BUMPT cells with NAT10 siRNA and a Spred2 overexpression plasmid. The findings indicated that NAT10 knockdown substantially suppressed TGF-β1-induced expression of fibrotic markers (Figure 5E-F) and reduced activation of the JNK and ERK/TGF-β axis (Figure 5G-H). This inhibitory effect was partially restored following Spred2 overexpression. Collectively, these observations indicate that Spred2 functions as a NAT10 downstream effector and contributes to TGF-β1-induced fibrotic responses in renal tubular epithelial cells through JNK and ERK/TGF-β signaling axis activation. To quantify the phosphorylation status of ERK and JNK, we performed phosphorylation-targeted ELISA to measure the relative ratio of phosphorylated protein to total protein across all experimental groups (Figure 5I). Consistent with the above immunoblotting results, NAT10 silencing markedly decreased the phosphorylation levels of ERK upon TGF-β1 treatment. These ELISA data confirm that NAT10 primarily drives fibrotic signaling through Spred2 mRNA stabilization, while also suggesting the existence of other Spred2-independent downstream pathways. Overall, these observations indicate that Spred2 serves as a downstream effector of NAT10 and contributes to TGF-β1-induced fibrotic responses via activation of the JNK and ERK/TGF-β signaling axes.

### T-NAT10-KO attenuates UUO-induced TIF by suppressing the Spred2/JNK/ERK/TGF-β pathway

To examine NAT10's *in vivo* function in renal fibrosis, renal tubular epithelial cell-specific NAT10 knockout (T-NAT10-KO) mice were created. The breeding strategy is presented in Figure 6A. Mice carrying floxed NAT10 alleles (NAT10^f/f^) underwent crossbreeding with Cdh16-Cre transgenic mice to accomplish tubular epithelial-specific NAT10 deletion. Genotyping was conducted using a three-primer PCR approach. T-NAT10-KO mice were identified by (1) amplification of a 356 bp fragment corresponding to the floxed allele, (2) absence or marked reduction of the 302 bp fragment representing the wild-type allele, and (3) amplification of a 420 bp fragment specific for Cre recombinase (Figure 6B). To assess NAT10 deficiency effects on renal fibrosis, age-matched male T-NAT10-WT and T-NAT10-KO littermates were arbitrarily assigned to sham or UUO surgery groups, with kidney tissues collected 7 days post-surgery. Histological examination using H&E and MTS demonstrated that T-NAT10-KO mice displayed markedly diminished tubular injury and collagen accumulation compared with T-NAT10-WT mice following UUO surgery (Figure 6C-E). Immunohistochemical staining further indicated that T-NAT10-KO significantly diminished UUO-induced elevation of FN, Collagen I, and α-SMA in kidney tissues (Figure 6F-G). Immunoblot analysis revealed that T-NAT10-KO markedly inhibited UUO-induced Spred2 and TGF-β expression, diminished JNK and ERK signaling activation, and reduced FN, Collagen I and III, and α-SMA protein levels (Figure 6H-K). Collectively, these observations indicate that T-NAT10-KO alleviates UUO-induced TIF through inhibiting the Spred2/JNK and ERK/TGF-β signaling axis.

### T-NAT10-KO ameliorates FA-induced renal fibrosis by inactivation of the Spred2/JNK and ERK/TGF-β axis

To further validate the role of NAT10 in a different model, we used the FA-induced nephropathy model. Wild-type mice received intraperitoneal FA injections (250 mg/kg) for five consecutive days, with kidney tissue collection occurring at days 0, 3, 7, and 14 following injection. H&E staining demonstrated progressive tubular injury over time, whereas MTS revealed a time-dependent increase in interstitial collagen deposition (Figure 7A-C). Consistently, WB analysis revealed that NAT10 and fibrotic marker proteins (Collagen I, Collagen III, and FN) increased progressively during this period (Figure 7D-E). Furthermore, age-matched male littermates of T-NAT10-WT and T-NAT10-KO mice were randomly allocated to control (saline) or model groups (FA, 250 mg/kg for 5 consecutive days), and kidney tissues were collected 14 days after the final injection. H&E and MTS revealed that FA-induced tubular injury and collagen deposition were markedly reduced in T-NAT10-KO mice compared with T-NAT10-WT mice (Figure 7F-H). Immunohistochemical staining further demonstrated that T-NAT10-KO significantly decreased FA-induced FN, Collagen I, and α-SMA expression in kidney tissues (Figure 7I-J). Immunoblot analysis showed that T-NAT10-KO markedly suppressed FA-induced Spred2 expression, reduced JNK and ERK signaling activation, and decreased protein levels of TGF-β, FN, Collagen I, Collagen III, and α-SMA (Figure 7K-N). In conclusion, T-NAT10-KO reduces FA-induced renal TIF by inhibiting the Spred2/JNK and ERK/TGF-β axis, further supporting the pro-fibrotic role of NAT10.

### 5-Fluorouridine suppresses TGF-β1-induced fibrotic marker expression in BUMPT cells

Targeting key regulatory genes with small-molecule compounds is an effective therapeutic strategy.5-Fluorouridine is a potent inhibitor of RNA modification that interacts with total and poly(A) RNA, displaying anti-proliferative properties and inducing apoptosis^12^. Structural prediction analysis in this study suggested a potential interaction between 5-Fluorouridine and NAT10 (Figure 8A). Dot blot analysis further demonstrated that treatment with 20 μM 5-Fluorouridine for 24 h markedly reduced global ac4C levels in BUMPT cells compared with remodeling (a NAT10 inhibitor) (Figure 8B), and this result was further validated by western blotting (Figure 8C-D). Additional IC50 assays were performed to evaluate its cytotoxicity, and the results indicated that the inhibitory efficacy of 5-Fluorouridine was superior to that of remodelin(Figure 8E). To assess the influence of 5-Fluorouridine on NAT10 and fibrotic marker expression, time-course and dose-response experiments were conducted in BUMPT cells. Immunoblot analysis showed that treatment with 5-Fluorouridine decreased NAT10 and fibrotic markers (FN, Collagen I, Collagen III) in a time-dependent manner (0, 6, 12, and 24 h) (Figure 8F-G). Additionally, 5-Fluorouridine treatment at 5, 10, and 20 μM concentrations showed dose-dependent decreases in these protein expressions (Figure 8H-I).

To further evaluate 5-Fluorouridine's impact under TGF-β1-induced fibrotic conditions, BUMPT cells underwent pretreatment with 10 μM 5-Fluorouridine for 24 h, succeeded by TGF-β1 (5 ng/mL) stimulation for another 24 h. Immunoblot analysis revealed that TGF-β1 stimulation substantially enhanced NAT10 and fibrotic marker (FN, Collagen I, Collagen III) expression, whereas 5-Fluorouridine treatment significantly diminished this increase (Figure 8J-M). To further verify the target specificity of 5-Fluorouridine, rescue experiments were performed by co-transfecting BUMPT cells with a NAT10 overexpression plasmid and 5-Fluorouridine. The results demonstrated that, even under NAT10 overexpression, 5-Fluorouridine effectively suppressed TGF-β1-induced fibrotic marker expression (Figure 8N-O) and attenuated activation of the JNK and ERK/TGF-β signaling axis (Figure 8P-Q). Together, these findings indicate that 5-Fluorouridine attenuates TGF-β1-induced fibrotic responses *in vitro*, likely through suppression of NAT10 expression and activity.

### 5-Fluorouridine attenuates UUO-induced renal TIF targeting NAT10

We next examined the *in vivo* effect of 5-Fluorouridine on renal TIF.We first assessed its time- and dose-dependent efficacy.C57 mice were intraperitoneally administered 5-Fluorouridine, and kidney tissues were harvested at 0, 12, 24, and 72 h after injection. Immunoblot analysis indicated a time-dependent decrease in NAT10 and fibrotic markers (FN, Collagen I, Collagen III) following 5-Fluorouridine treatment (Figure 9A-B). Subsequently, mice received escalating 5-Fluorouridine doses (2.5, 5, and 10 mg/kg), showing dose-dependent decreases in fibrosis-associated protein expression levels (Figure 9C-D). Based on these observations, the therapeutic potential of 5-Fluorouridine in UUO-induced renal fibrosis was further assessed. Age-matched male littermate mice were randomly assigned to control or UUO model groups and administered intraperitoneal injections of 5-Fluorouridine (10 mg/kg) or vehicle every 72 hours. Histological analysis via H&E and MTS demonstrated that 5-Fluorouridine markedly alleviated UUO-induced renal tissue injury and collagen accumulation (Figure 9E-G). Immunohistochemical staining additionally demonstrated that 5-Fluorouridine treatment markedly decreased UUO-induced Collagen I, α-SMA, and FN expression in renal tissues (Figure 9H-I). Immunoblot analysis also showed that 5-Fluorouridine markedly suppressed UUO-induced expression of Spred2, p-JNK, p-ERK, TGF-β, and fibrotic markers (FN, Collagen I, Collagen III, α-SMA) (Figure 9J-M). Collectively, these findings demonstrate that 5-Fluorouridine efficiently mitigates UUO-induced renal TIF through NAT10 targeting and inhibition of the Spred2/JNK and ERK/TGF-β1 signaling axis.

### 5-Fluorouridine ameliorates FA-induced renal TIF via targeting NAT10

The FA-induced nephropathy model, widely recognized as a classical model representing the transition from AKI to CKD, demonstrates strong clinical relevance for particular patient populations^13^. However, the potential role of 5-Fluorouridine in FA-induced renal TIF remains insufficiently characterized. Renal fibrosis was established via intraperitoneal FA administration (250 mg/kg) for five consecutive days, following which mice underwent random assignment to control or model groups receiving intraperitoneal treatment with or without 5-Fluorouridine (10 mg/kg) every72 hours for 14 days. Histopathological examination via H&E and MTS revealed that 5-Fluorouridine administration substantially reduced FA-induced renal tissue damage and collagen deposition (Figure 10A-C). Immunohistochemical staining additionally showed that 5-Fluorouridine treatment significantly decreased Collagen I, α-SMA, and FN expression in kidney tissues from FA-treated mice (Figure 10D-E). Immunoblot examination also revealed that FA-induced expression of Spred2, p-JNK, p-ERK, TGF-β, and fibrotic markers (FN, Collagen I, Collagen III, and α-SMA) was substantially diminished after 5-Fluorouridine administration (Figure 10F-I). Collectively, these observations demonstrate that 5-Fluorouridine efficiently mitigates FA-induced renal TIF through targeting NAT10 and suppressing the Spred2/JNK and ERK/TGF-β1 signaling axis, further supporting its anti-fibrotic potential.

## Discussion

Previous studies have implicated the RNA acetyltransferase NAT10 in myocardial and pulmonary fibrosis. The present study extends these findings by showing that NAT10 also promotes renal tubulointerstitial fibrosis (TIF) induced by UUO and FA. Mechanistically, the transcription factor p53 directly binds to the NAT10 promoter, enhancing its transcriptional activity. This, in turn, promotes Spred2 expression through ac4C-mediated acetylation, leading to activation of the JNK and ERK/TGF-β signaling axes. Furthermore, 5-Fluorouridine, a potent ribozyme self-cleavage inhibitor, was identified as a novel NAT10-targeting compound that attenuates UUO- and FA-induced renal TIF. Collectively, these findings reveal a previously unrecognized mechanism by which NAT10 promotes renal fibrosis and identify a potential therapeutic compound targeting NAT10 for TIF intervention.

The diverse regulatory roles of NAT10 in kidney diseases are beginning to emerge. In renal cell carcinoma, Miao *et al*. demonstrated that NAT10 facilitates tumor progression and lymphangiogenesis through the ANKZF1/YWHAE/YAP1 signaling axis^14^, whereas Sun *et al*. reported that NAT10 promotes malignant phenotypes through NFE2L3/AKT/GSK3β pathway activation^15^. In AKI contexts, Wang *et al*. showed that NAT10-mediated ac4C modification aggravates renal inflammatory damage by enhancing the CCL2/CXCL1 chemokine signaling axis^16^. Building on these observations, our study is the first to demonstrate that NAT10 mediates TIF induced by both UUO and FA.

Little information is currently available regarding the regulatory mechanism responsible for NAT10 upregulation. The tumor suppressor p53 participates in diverse biological processes, encompassing cell proliferation, apoptosis, senescence, and metabolic regulation^17^-^20^. Recent studies have further clarified its important role in AKI and subsequent renal repair through mechanisms involving apoptosis, cell cycle arrest, and autophagy^21^-^23^. This investigation extends p53's mechanistic framework to the epitranscriptomic level by revealing that p53 functions as a transcription factor with the capacity to directly bind the NAT10 promoter and augment its transcriptional activity, thus establishing p53 as an upstream regulator of NAT10. But the mechanisms upstream of p53 itself in UUO/FA models remain elusive. Hypoxia, oxidative stress, and DNA damage may activate p53 via kinases such as ATM/ATR. Elucidating these upstream events may provide additional targets for early intervention in fibrosis. Subsequently, NAT10 specifically increases the expression of its downstream target gene Spred2 through ac4C-mediated acetylation. Spred2, a negative regulator of Ras/ERK signaling, has traditionally been reported to inhibit ERK activation^24^-^27^. However, the current findings indicate that, under pathological conditions induced by UUO and FA injury, increased Spred2 expression paradoxically facilitates sustained activation of the JNK and ERK axis. These observations suggest the presence of a context-dependent functional transition of Spred2 during renal fibrosis. The underlying mechanisms remain to be elucidated and may involve interactions with other stress-responsive signaling pathways or regulatory events mediated by post-translational modifications. The JNK and ERK signaling pathways are well-established mediators of pro-fibrotic and pro-inflammatory responses, including TGF-β signaling^28^,^29^. our results indicate that the p53/NAT10/Spred2 axis acts as an upstream event responsible for persistent JNK/ERK activation in UUO- and FA-induced fibrosis models.

Inhibition of NAT10 has been proposed as a therapeutic strategy for various diseases. Remodelin, a well-characterized NAT10 inhibitor, has been extensively investigated in cancer immunotherapy and Hutchinson-Gilford progeria syndrome, among other pathological conditions^30^. In this study, systematic validation using both* in vitro* cellular experiments and *in vivo* animal models, encompassing UUO and FA-induced nephropathy models, demonstrates for the first time that 5-Fluorouridine functions as a novel and effective NAT10 inhibitor with significant therapeutic efficacy against renal fibrosis. Although 5-Fluorouridine effectively targets NAT10, its potential toxicity as a 5-FU analog—including myelosuppression and gastrointestinal reactions—should not be overlooked. Future studies should assess its therapeutic window and long-term safety, and explore kidney-targeted delivery strategies to reduce systemic exposure. Future investigations will further determine the precise binding site and binding affinity of 5-Fluorouridine to NAT10 and will also clarify the molecular mechanism responsible for the reduction of NAT10 protein levels induced by 5-Fluorouridine.

In conclusion, we demonstrate for the first time that p53-induced NAT10 mediates renal TIF progression both *in vitro* and *in vivo*. Mechanistically, NAT10 promotes Spred2 expression via ac4C-mediated acetylation, thereby activating the JNK and ERK/TGF-β signaling axis. Notably, we identify 5-Fluorouridine as an effective NAT10 inhibitor that attenuates TIF in UUO and FA models. Together, these findings indicate that the NAT10/Spred2/JNK and ERK/TGF-β axis contributes to the development of renal TIF, and that targeting NAT10 with 5-Fluorouridine represents a promising epitranscriptomic strategy for therapy. Specifically, this strategy targets NAT10 to inhibit ac4C RNA modification, reduce Spred2 mRNA stability, suppress JNK and ERK/TGF-β signaling, and ultimately mitigate TIF. Of note, this study focused on NAT10 in tubular epithelial cells; however, renal fibrosis involves multiple cell types, including fibroblasts and macrophages. The role of NAT10 in these non-tubular cells remains unclear. Therefore, future studies using cell-specific NAT10 knockout mice are needed to fully clarify the regulatory network of NAT10 in TIF and to expand the therapeutic applicability of this strategy, providing a solid theoretical basis for its clinical translation.

## Materials and methods

### Antibodies and reagents

The primary antibodies employed in this investigation were as follows: anti-FN (Abcam, ab2413), anti-NAT10 (Abcam, ab194297), and anti-ac4C (Abcam, ab252215) were obtained from Abcam (Cambridge, UK); anti-Collagen I (Proteintech, 67288-1-Ig), anti-α-SMA (Proteintech, 14395-1-AP), anti-β-Tubulin (Proteintech, 10094-1-AP), and anti-GAPDH (Proteintech, 60004-1-Ig) were acquired from Proteintech Group (Rosemont, IL, USA); anti-Collagen III (Affinity, AF5457) and anti-Spred2 (Affinity, DF13707) were obtained from Affinity Biosciences; anti-p53 (Cell Signaling Technology, 2524T) were procured from Cell Signaling Technology (Danvers, MA, USA). Secondary antibodies were procured from ThermoFisher Scientific (Waltham, MA, USA). Protein signals were visualized employing an enhanced chemiluminescence (ECL) detection kit (Tanon, Shanghai, China; #180-5001). NAT10 siRNA was obtained from RiboBio (Guangzhou, China), whereas Spred2 siRNA was supplied by Hema Biotech (Zhejiang, China). Plasmids were synthesized by Tsingke (Beijing, China). 5-Fluorouridine (HY-107856) was procured from MCE (Shanghai, China).

### Cell culture and TGF-β1 treatment

BUMPT cells, an immortalized mouse proximal tubular epithelial cell line, were maintained in Dulbecco's Modified Eagle Medium (DMEM; Sigma-Aldrich, St. Louis, MO, USA) comprising 10% fetal bovine serum (FBS) and 1% penicillin-streptomycin (Gibco, 15140163). Cells underwent cultivation at 37°C within a humidified incubator with 5% CO₂. After achieving 80-90% confluence, cellular subculturing occurred at a 1:3 ratio. Once cellular confluence reached approximately 70%, overnight serum deprivation was performed, subsequently followed by stimulation using 5 ng/mL recombinant TGF-β1 (R&D Systems, Minneapolis, MN, USA) for 6, 12, or 24 h. This concentration selection was established according to previously published studies^31^,^32^. Transient transfection of NAT10 siRNA and Spred2 siRNA was carried out using Lipofectamine 2000 (Invitrogen) when cells reached approximately 50% confluence. The generation of NAT10 stable overexpression cell lines was described in a previous study^31^.

### Animal models

To examine the role of NAT10 in renal TIF, renal tubular epithelial cell-specific NAT10 knockout (T-NAT10-KO) mice were generated. The unilateral ureteral obstruction (UUO) model was created by ligating the left ureter, with animal sacrifice occurring on postoperative day 7, as described previously^33^,^34^. The FA-induced nephropathy model was established via intraperitoneal FA administration (250 mg/kg) for five consecutive days, with kidney tissue collection performed 14 days following the last injection. All mice were housed under a 12-hour light/dark cycle with unrestricted access to food and water. All animal experimental procedures were sanctioned by the Institutional Ethics Committee of The Second Xiangya Hospital, Central South University (Approval No. 20260730).

### Histological and immunohistochemical analysis

Renal tissues underwent fixation in 4% paraformaldehyde, followed by paraffin embedding and sectioning into 4 μm-thick slices. Hematoxylin and eosin (H&E) staining was executed to examine tubular and glomerular morphology along with tissue injury. Masson trichrome staining (MTS) was utilized to examine collagen deposition and distribution within the renal interstitium, following previously described protocols^35^,^36^. Immunohistochemical staining was executed employing primary antibodies targeting Collagen I (1:100), FN (1:100), α-SMA (1:100), and NAT10 (1:100), according to established procedures^37^. Subsequently, the stained sections were examined and photographed utilizing an Olympus fluorescence microscope.

### Reverse transcription quantitative PCR (RT-qPCR)

Total RNA extraction from BUMPT cells or kidney tissues utilizing Trizol reagent (Invitrogen, Carlsbad, CA, USA). Complementary DNA synthesis was performed utilizing the PrimeScript RT reagent kit with gDNA Eraser (TaKaRa, RR037A) per the supplier's protocols, as described previously^35^. Quantitative PCR amplification was executed using TB Green Premix Ex Taq (TaKaRa, RR820A) on a LightCycler 96 system (Roche). The primer sequences employed were as follows: NAT10 forward: GGAGCATCTGGAGGCACTTT, reverse: CAGGGACAGGTCTCCTAGCT; Spred2 forward: ACCTCCTCTTCCACTCTCCATAA, reverse: CGTAGTCGGCTCCCTCTTCTG. Relative gene expression levels were ascertained employing the 2^⁻ΔΔCT^ methodology, whereas absolute quantification was performed through standard curve analysis.

### Western blot (WB) analysis

WB analysis followed previously established protocols^38^. Total proteins were procured from cellular samples or renal tissues utilizing radio-immunoprecipitation assay lysis buffer, separated via sodium dodecyl sulfate-polyacrylamide gel electrophoresis, and then transferred to polyvinylidene fluoride membranes. Membranes underwent blocking with 5% non-fat milk for 2 h at ambient conditions, succeeded by overnight incubation at 4°C with primary antibodies: NAT10 (1:1000), FN (1:1000), Collagen I (1:1000), Collagen III (1:1000), Spred2 (1:1000), p53 (1:1000), p-JNK (1:1000), JNK (1:1000), p-ERK (1:1000), ERK (1:1000), TGF-β (1:1000), β-Tubulin (1:5000), and GAPDH (1:5000). After primary antibody incubation, membranes received treatment with horseradish peroxidase-conjugated secondary antibodies for 1 h at ambient conditions. Protein signals underwent detection employing an ECL detection kit (Tanon, Shanghai, China) and visualization with a chemiluminescence imaging system (Tanon 4800). Quantitative densitometric analysis of band intensities was executed using ImageJ software.

### RNA stability assay

BUMPT cells stably expressing scramble-shRNA or NAT10-shRNA were exposed to actinomycin D (5 μg/mL; Sigma, SBR00013). Total RNA was harvested at specified time intervals, and Spred2 mRNA abundance was quantified by RT-qPCR to evaluate mRNA decay kinetics.

### Ac4C dot blot assay

Total RNA was procured from cells and was normalized to equal concentrations. RNA samples were combined with 20× SSC buffer and 37% formaldehyde at a ratio of 3:2, denatured at 95°C for 5 min, and subsequently applied onto nitrocellulose membranes (1 μL per spot). The RNA was crosslinked to the membrane by ultraviolet irradiation at 302 nm. Membranes underwent blocking with 5% non-fat milk for 2 h at ambient conditions, succeeded by overnight incubation at 4°C with anti-Ac4C antibody, then exposed to the corresponding secondary antibody for 1 h at ambient conditions. After comprehensive rinsing with PBST, membranes underwent treatment with ECL reagent, and chemiluminescent signals were captured utilizing a chemiluminescence imaging system.

### Immunofluorescence staining

BUMPT cells grown on coverslips underwent fixation with 4% paraformaldehyde, permeabilization using 0.1% Triton X-100, and blocking with 5% bovine serum albumin. Cells underwent overnight incubation at 4°C with anti-NAT10 antibody (1:100), then received 1 h incubation with fluorescence-conjugated secondary antibody at 37°C under dark conditions. Nuclear counterstaining utilized DAPI for 3-5 min. Subsequently, fluorescent signals were detected utilizing a fluorescence microscope.

### Chromatin immunoprecipitation (ChIP) assay

ChIP assays were executed utilizing a ChIP kit (Millipore, Billerica, MA, USA) per the supplier's protocols. Chromatin fragments underwent immunoprecipitation with an anti-p53 antibody, while normal mouse IgG served as a negative control. The primer sequences employed in ChIP analysis were as follows: P53 (1) forward: TTTGTACATGCACAATTCACAGT, reverse: ACTACAATCATTGGAAGGTCATGT; P53 (2) forward: AGGGAACTGATGGTCCTACA, reverse: AGTCATCAATGGCATGCTACCT.

### RNA immunoprecipitation (RIP) assay

RIP assays were executed employing an RIP kit (Millipore) per the supplier's protocol. RNA-protein complexes were immunoprecipitated utilizing an anti-NAT10 antibody, while IgG was utilized as a negative control. Subsequently, the co-precipitated RNA underwent extraction and RT-qPCR analysis to evaluate Spred2 mRNA enrichment.

### Dual-luciferase reporter assay

BUMPT cells underwent co-transfection with wild-type or mutant Spred2 luciferase reporter plasmids alongside a NAT10 overexpression plasmid via Lipofectamine 2000. After 48 h post-transfection, luciferase activities were assessed utilizing the Dual-Luciferase Reporter Assay System (Promega, Madison, WI, USA) per the supplier's protocol. Renilla luciferase activity functioned as the internal normalization control.

### Statistical analysis

All data are presented as mean ± standard deviation (SD). Two-tailed Student's t-test was adopted for comparisons between two independent groups. For comparisons involving three or more groups, one-way analysis of variance (ANOVA) with Bonferroni post hoc correction was applied to correct for multiple comparisons. Nonparametric datasets were analyzed using the Kruskal-Wallis test. Differences with P < 0.05 were considered statistically significant. In all figure legends, asterisk (*) and hash symbol (#) both represent P < 0.05 after multiple comparison correction, with specific comparison objects annotated separately in each figure legend.

## Figures and Tables

**Figure 1:**
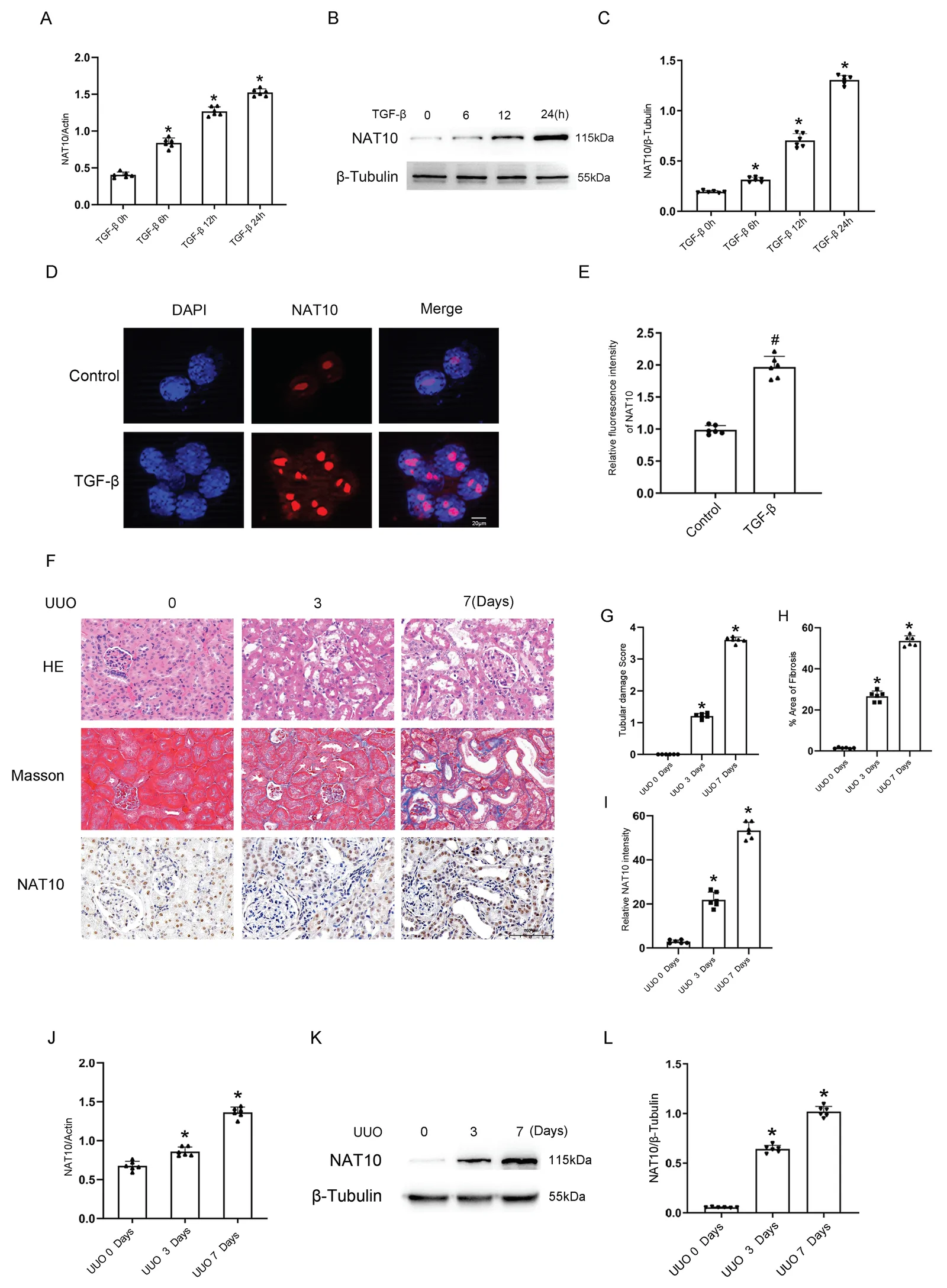
** NAT10 expression shows upregulation in BUMPT cells after TGF-β1 exposure and in kidneys from UUO mice.** (A-C) RT-qPCR and immunoblot analysis assessed NAT10 mRNA and protein levels in BUMPT cells following TGF-β1 treatment, with densitometric quantification. (D-E) Immunofluorescence staining demonstrates NAT10 expression patterns and subcellular localization in BUMPT cells after TGF-β1 exposure. Original magnification ×600; Scale bar = 20 μm. Data are presented as mean ± SD (n = 6). #*P < 0.05* TGF-βvs. control group. (F) Representative micrographs show H&E staining, MTS revealing fibrotic regions, and immunohistochemical staining for NAT10 in kidney sections from UUO mice at specified time points. Scale bar = 100 μm. (G) Quantitative evaluation of tubular injury scores. (H-I) Correlation analysis between the percentage of NAT10-positive areas and the extent of fibrotic regions. (J-L) Immunoblot analysis showing NAT10 protein abundance in kidney tissues collected from mice at days 0, 3, and 7 following UUO surgery, with β-Tubulin utilized as the internal loading control, accompanied by densitometric quantification. Data are denoted as mean ± SD (n = 6). *P < 0.05 vs. sham or control group.

**Figure 2:**
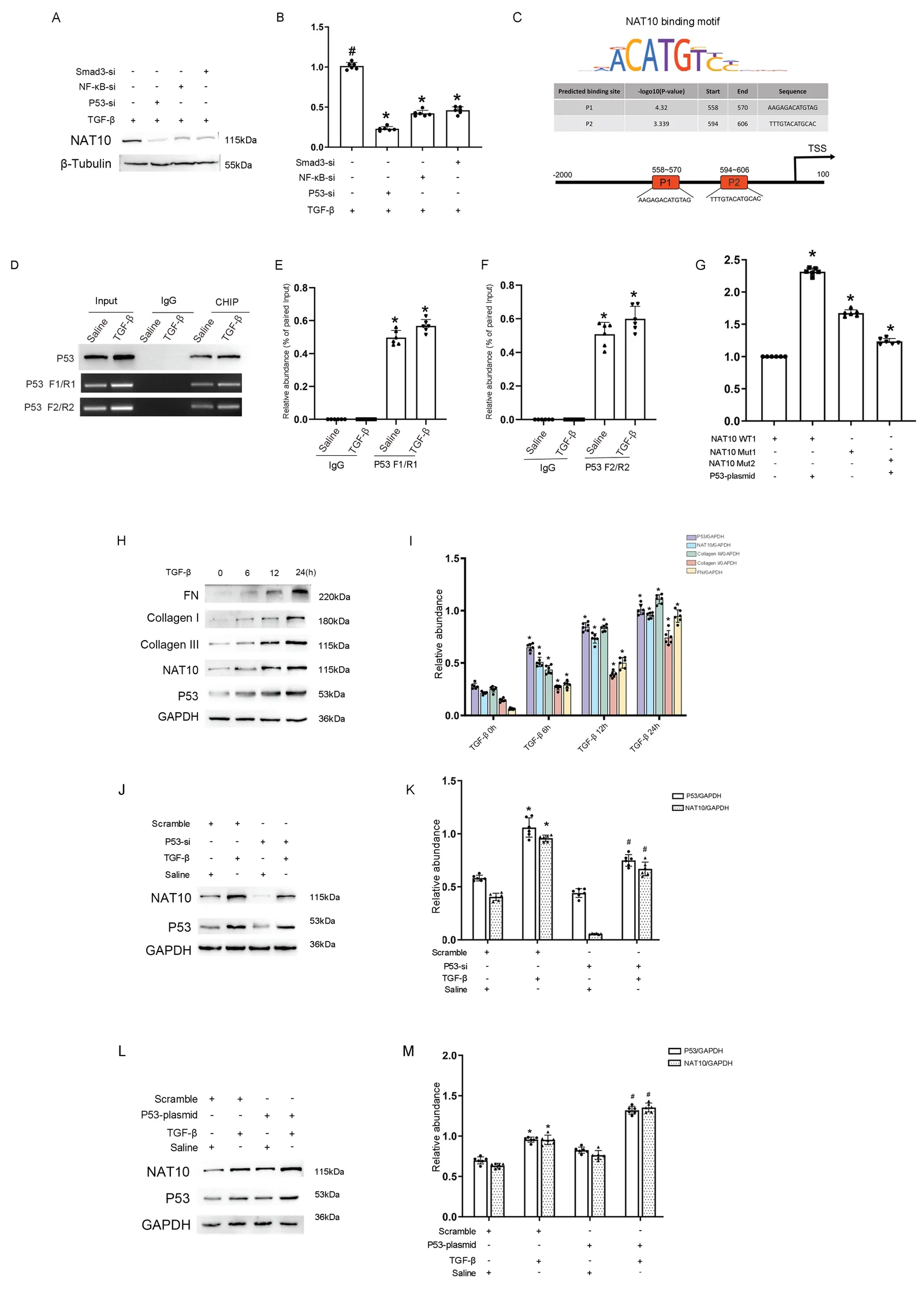
** p53 functions as a transcription factor to promote TGF-β1-induced fibrosis in BUMPT cells by regulating NAT10 expression.** BUMPT cells underwent transfection with p53 siRNA or a p53 overexpression plasmid, succeeded by exposure to 5 ng/mL TGF-β1 or no treatment for 24 h. (A-B) Immunoblot analysis with corresponding densitometric quantification comparing the effects of p53, NFκB, and Smad3 knockdown on NAT10 expression in BUMPT cells. p53 knockdown resulted in the most pronounced reduction in NAT10 expression.(C) Bioinformatic prediction using MoLoTool (http://molotool.autosome.org) identified putative p53-binding motifs in the mouse NAT10 gene promoter region. (D) ChIP assays performed using chromatin isolated from BUMPT cells verified the direct association of p53 with the NAT10 promoter region. (E-F)Quantitative verification of p53 binding to NAT10 promoter via ImageJ grayscale normalization of ChIP products. IgG control showed negligible non-specific binding. Relative binding proportion was normalized against paired Input DNA for each group. (G)Dual-luciferase reporter assays demonstrated that p53 overexpression significantly enhanced luciferase activity in the wild-type NAT10 plasmid, whereas this increase was markedly attenuated in the mutant p53 plasmid with disrupted binding sites.(H-I) WB analysis with corresponding densitometric quantification demonstrating the protein levels of p53, NAT10, and fibrotic markers (FN, Col I, Col III) in BUMPT cells in the presence or absence of TGF-β1 stimulation. (J-K) WB analysis of p53 and NAT10 protein expression in BUMPT cells exposed to TGF-β1 and/or p53 siRNA, with β-Tubulin utilized as the loading control, accompanied by densitometric quantification. (L-M) WB analysis of p53 and NAT10 protein expression in BUMPT cells exposed to TGF-β1 and/or p53 overexpression, with β-Tubulin employed as the loading control, together with densitometric quantification. Data are denoted as mean ± SD (n = 6). *p< 0.05, TGF-β with Scramble group vs. Saline with Scramble group; #*P* < 0.05 TGF-β + P53 siRNA group or TGF-β1 + P53 plasmid group vs. TGF-βwith Scramble group.

**Figure 3:**
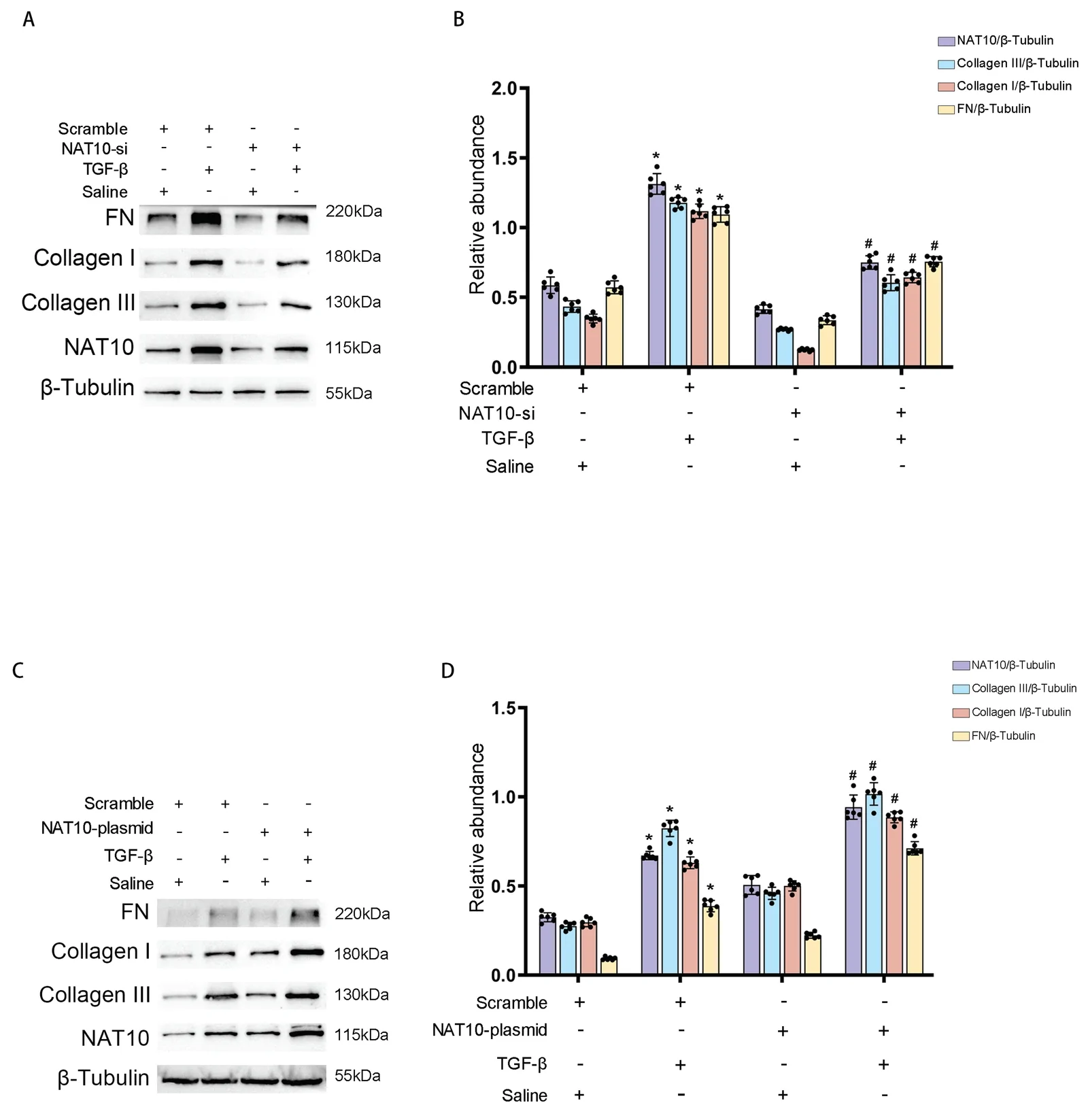
** NAT10-mediated TGF-β1-induced FN, Collagen I, and Collagen III expression in BUMPT cells.** BUMPT cells were transfected with NAT10 siRNA or an NAT10 overexpression plasmid, then received treatment with 5 ng/mL TGF-β1 or remained untreated for 24 h. (A-B) WB analysis with corresponding densitometric quantification examining NAT10 and fibrotic markers (FN, Col I, Col III) in BUMPT cells following transfection with NAT10 siRNA or control siRNA, under conditions with or without TGF-β1 stimulation. (C-D) WB analysis accompanied by densitometric quantification evaluating NAT10 and fibrotic markers (FN, Col I, Col III) in BUMPT cells transfected with an NAT10 overexpression plasmid or empty vector, with or without TGF-β1 stimulation. Data are denoted as mean ± SD (n = 6). *p< 0.05, TGF-β with Scramble group vs. Saline with Scramble group; #*P < 0.05* TGF-β1 + NAT10 siRNA group or TGF-β1 + NAT10 plasmid group vs.TGF-βwith Scramble group.

**Figure 4:**
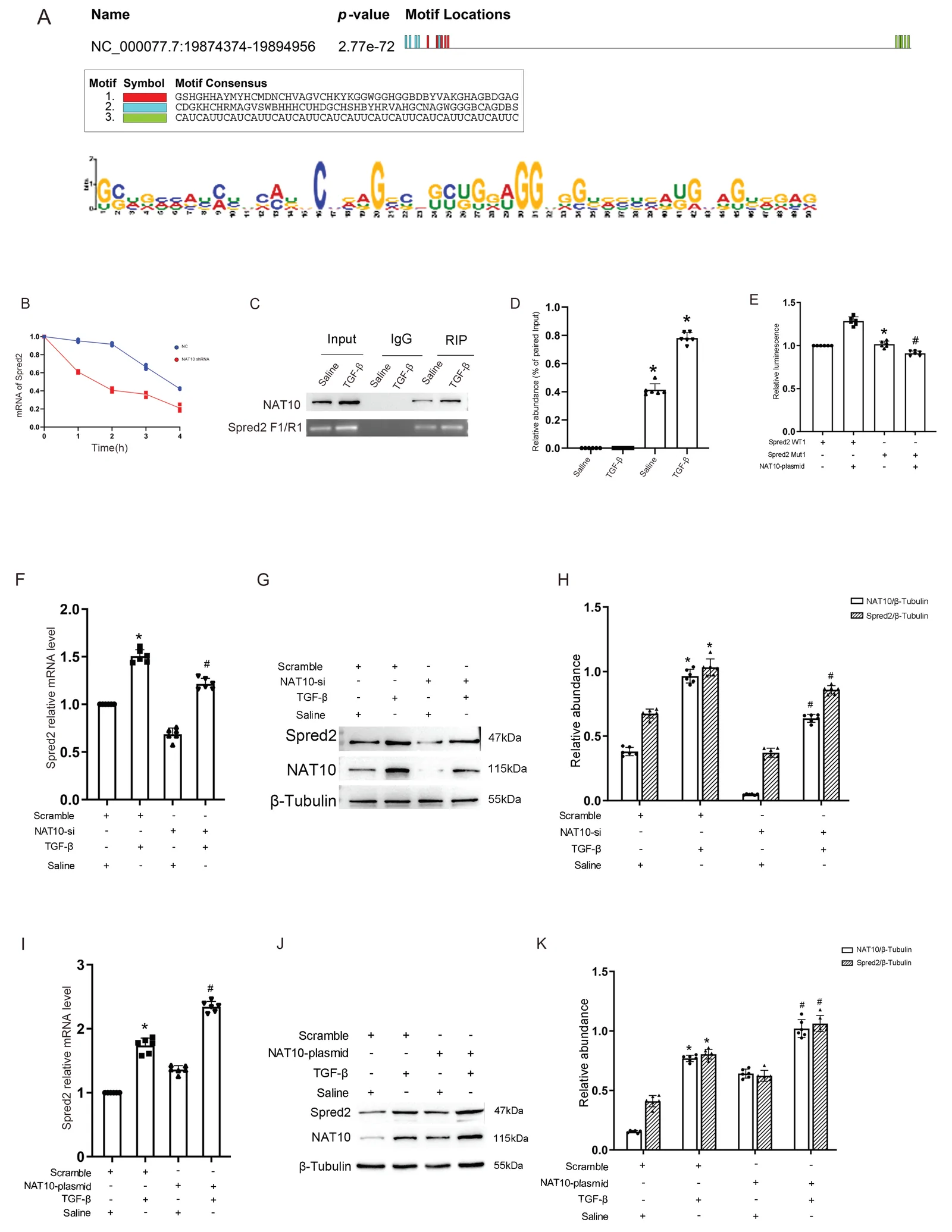
** NAT10 regulates Spred2 expression through acetylation-mediated mRNA stability.** (A) Bioinformatic prediction performed using MEME software (https://meme-suite.org), identifying potential NAT10-binding regions within Spred2 mRNA. (B) RT-qPCR analysis measuring Spred2 mRNA levels at the indicated time points in scramble-shRNA and NAT10-shRNA stably transfected BUMPT cells after treatment with actinomycin D (5 μg/mL). (C) RIP assay showing the interaction between NAT10 and Spred2 mRNA at predicted binding sites. (D)RIP coupled with western blot and grayscale semi-quantification. (E) Dual-luciferase reporter assay executed in cells co-transfected with NAT10 overexpression plasmid and wild-type or mutant Spred2 luciferase reporters. *P < 0.05 vs. Spred2-Mut group; #*P* < 0.05 vs. other groups. (F-H) RT-qPCR and WB analyses with densitometric quantification evaluating NAT10 and Spred2 expression in BUMPT cells following transfection with NAT10 siRNA or control siRNA, under conditions with or without TGF-β1 stimulation. (I-K) RT-qPCR and WB analyses with densitometric quantification assessed NAT10 and Spred2 expression in BUMPT cells after infection with NAT10 overexpression virus or control virus, with or without TGF-β1 stimulation. Data are denoted as mean ± SD (n = 6). *p< 0.05, TGF-β with Scramble group vs. Saline with Scramble group; #*P* < 0.05 TGF-β1 + NAT10 siRNA group or TGF-β1 + NAT10 plasmid group vs.TGF-βwith Scramble group.

**Figure 5:**
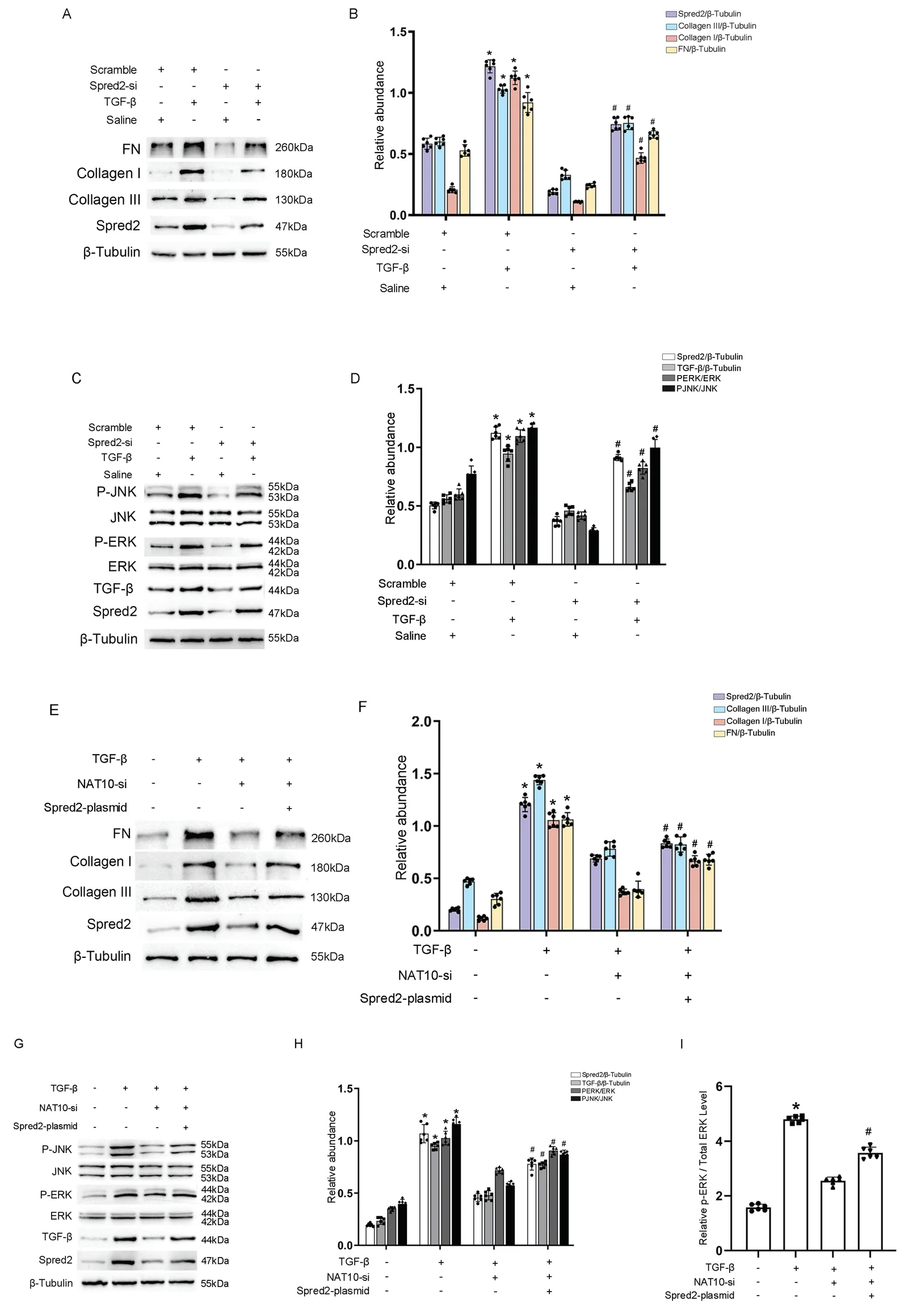
** Spred2 mediates TGF-β1-induced fibrotic marker expression and modulates the JNK/ERK/TGF-β signaling pathway.** (A-B) WB analysis with densitometric quantification examining Spred2 and fibrotic markers (FN, Col I, and Col III) in BUMPT cells following transfection with Spred2 siRNA or control siRNA, with or without TGF-β1 treatment (5 ng/mL, 24 h). (C-D) WB analysis with densitometric quantification examining JNK, p-JNK, ERK, p-ERK, and TGF-β protein expression in BUMPT cells under treatments specified in (A). (E-F) WB analysis with corresponding densitometric quantification examining Spred2 and fibrotic markers (FN, Collagen I, and Collagen III) in BUMPT cells co-transfected with NAT10 siRNA and/or Spred2 overexpression plasmid, succeeded by TGF-β1 treatment (5 ng/mL, 24 h). (G-H) WB analysis and densitometric quantification measuring JNK, p-JNK, ERK, p-ERK, and TGF-β expression in BUMPT cells subjected to treatments described in (E).(I) ELISA quantitative detection of relative phosphorylation level of P-ERK and ERK in rescue experiment groups. Data are denoted as mean ± SD (n = 6). *p< 0.05, TGF-β with Scramble group vs. Saline with Scramble group; TGF-β with NAT10 plasmid group or TGF-β with Spred2 siRNA group.

**Figure 6:**
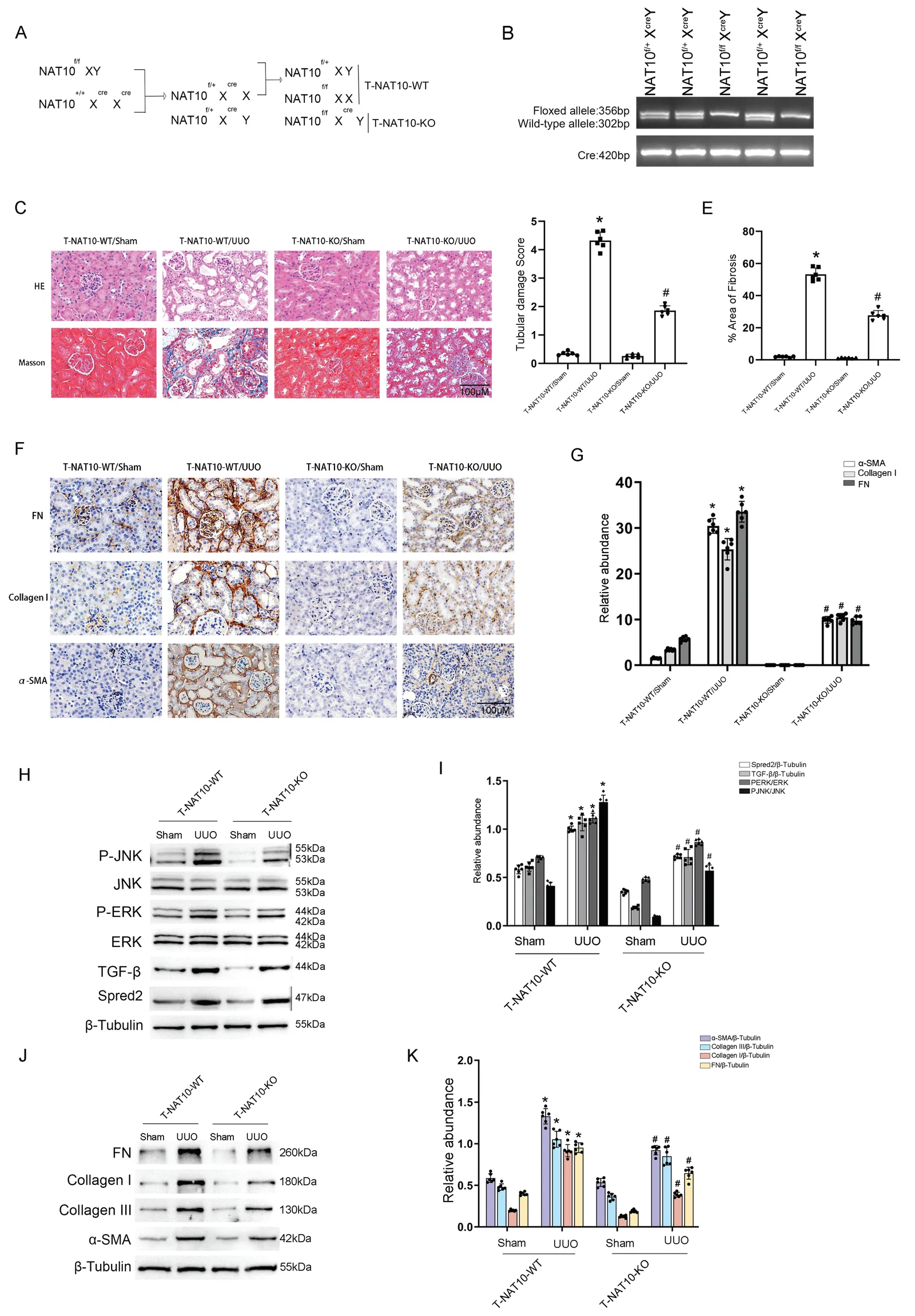
** Tubular-specific NAT10 knockout reduces UUO-induced renal fibrosis via suppression of Spred2/JNK and ERK/TGF-β signaling.** Age- and weight-matched male littermate T-NAT10-WT and T-NAT10-KO mice were randomly allocated to sham or UUO surgery groups. (A) Breeding approach employed for generating T-NAT10-KO mice. (B) PCR-based genotyping detects wild-type and floxed NAT10 alleles alongside the Cdh16-Cre transgene. (C) Representative micrographs showing H&E and MTS of kidney sections from the specified groups. Scale bar = 100 μm. (D) Quantitative assessment of tubular injury scores. (E) Quantification of fibrotic areas. (F-G) Immunohistochemical staining with corresponding quantification of fibrotic markers (FN, Collagen I, and α-SMA) in kidney sections from the indicated groups. Scale bar = 100 μm. (H-K) WB analysis with densitometric quantification evaluating Spred2, JNK, p-JNK, ERK, p-ERK, TGF-β, and fibrotic markers (FN, Collagen I, Collagen III, α-SMA) in kidney tissues derived from the indicated groups, with β-Tubulin utilized as the loading control. Data are denoted as mean ± SD (n = 6). *p< 0.05, UUO with NAT10-WT group vs. sham with NAT10-WT group; #p< 0.05, UUO with NAT10-KO group vs. sham with NAT10-KO group.

**Figure 7:**
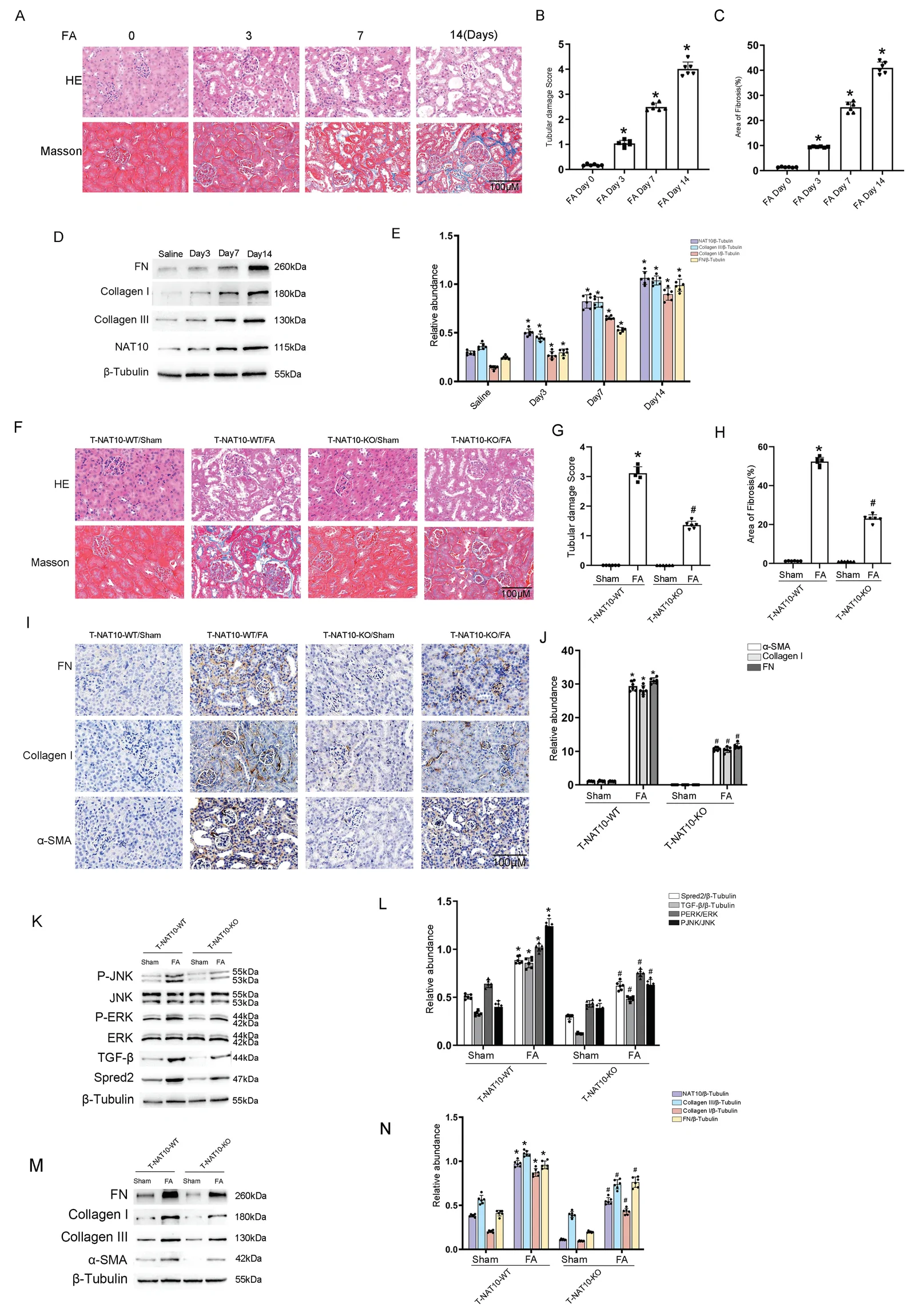
** T-NAT10-KO attenuates FA-induced renal fibrosis by inhibition of the Spred2/JNK and ERK/TGF-β pathway.** Age- and weight-matched littermate T-NAT10-WT and T-NAT10-KO mice were randomly allocated to saline or FA treatment groups. (A) Representative micrographs of H&E and MTS from kidney sections obtained from wild-type mice at designated time points (days 0, 3, 7, and 14) following FA administration (250 mg/kg for 5 consecutive days). Scale bar = 100 μm. (B) Quantitative evaluation of tubular injury scores. (C) Quantification of fibrotic areas. (D-E) WB analysis with densitometric quantification assessing NAT10 and fibrotic markers (Col I, Col III, FN) in kidney tissues collected at designated time points. (F) Representative micrographs of H&E and MTS of kidney sections derived from T-NAT10-WT and T-NAT10-KO mice treated with saline or FA. Scale bar = 100 μm. (G) Quantitative assessment of tubular injury scores. (H) Quantification of fibrotic areas. (I-J) Immunohistochemical staining with corresponding quantification of fibrotic markers (FN, Col I, and α-SMA) in kidney sections from the indicated groups. Scale bar = 100 μm. (K-N) WB analysis accompanied by densitometric quantification determining Spred2, JNK, p-JNK, ERK, p-ERK, TGF-β, and fibrotic markers (FN, Collagen I, Collagen III, α-SMA) in kidney tissues from the indicated groups, with β-Tubulin utilized as the loading control. Data are denoted as mean ± SD (n = 6). *p< 0.05, Folic acid with NAT10-WT group vs. sham with NAT10-WT group; #p< 0.05, Folic acid with NAT10-KO group vs. sham with NAT10-KO group.

**Figure 8:**
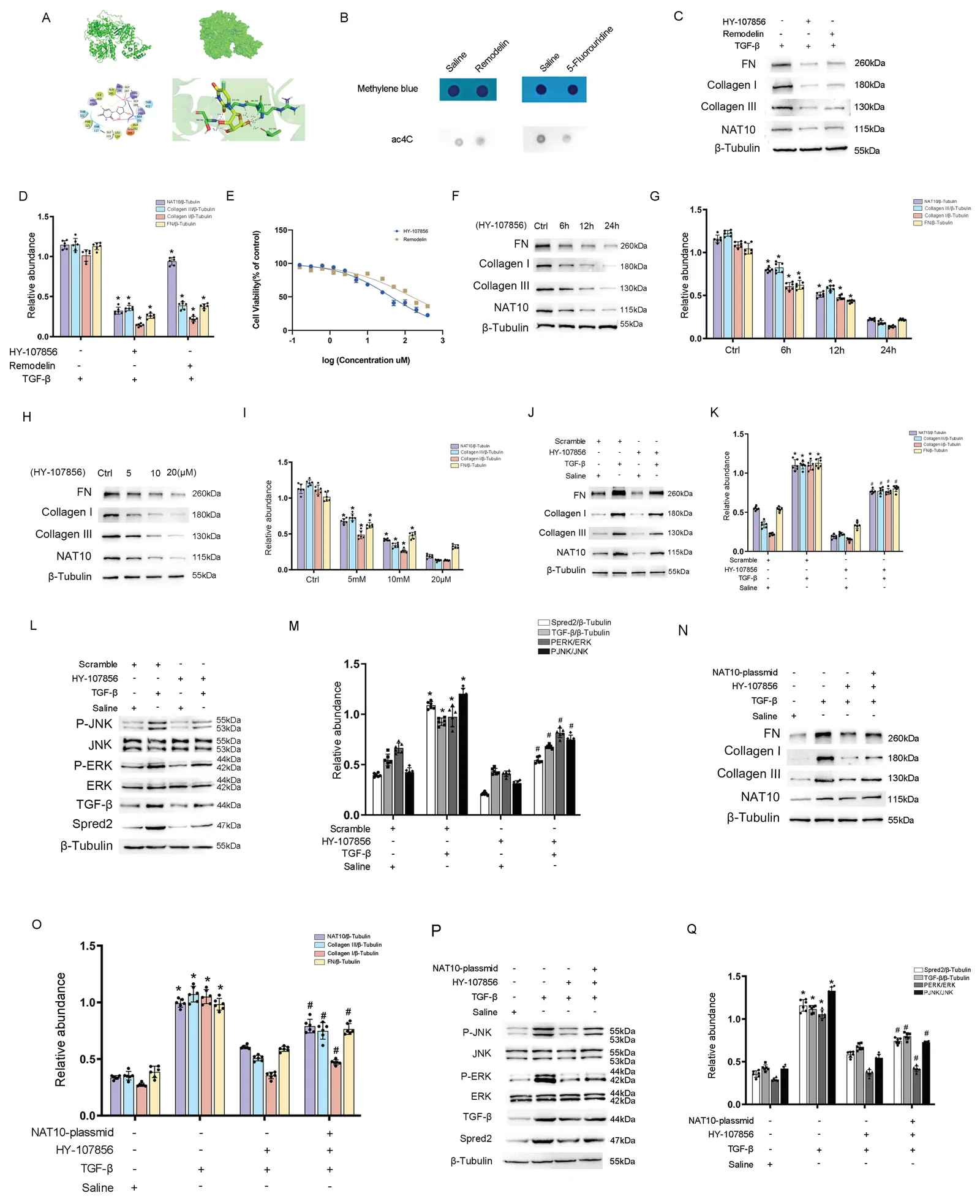
** 5-Fluorouridine attenuates TGF-β1-induced renal fibrosis in BUMPT cells. (A)** Structural modeling predicting the interaction between NAT10 and 5-Fluorouridine. (B) Dot blot analysis evaluating global ac4C levels in BUMPT cells exposed to saline or 5-Fluorouridine (20 μM, 24 h). (C-D) Immunoblot analysis with corresponding densitometric quantification comparing the effects of 5-Fluorouridine and remodeling on NAT10 and fibrotic marker (FN, Collagen I, and Collagen III) expression in BUMPT cells. (E) IC50 assays evaluating the cytotoxicity of 5-Fluorouridine and remodeling. The results indicated that the inhibitory efficacy of 5-Fluorouridine was superior to that of remodeling (F-G) WB analysis with corresponding densitometric quantification assessing NAT10 and fibrotic markers (FN, Collagen I, and Collagen III) in BUMPT cells exposed to 5-Fluorouridine for the indicated time intervals (0, 6, 12, and 24 h). (H-I) Immunoblot analysis with densitometric quantification assessing NAT10 and fibrotic markers (FN, Collagen I, and Collagen III) in BUMPT cells exposed to escalating 5-Fluorouridine concentrations (5, 10, and 20 μM) for 24 h. (J-M) Immunoblot analysis with densitometric quantification examining NAT10 and fibrotic markers (FN, Collagen I, Collagen III) in BUMPT cells pre-exposed to 5-Fluorouridine (20 μM, 24 h) followed by TGF-β1 stimulation (5 ng/mL, 24 h).(N-O) Immunoblot analysis with corresponding densitometric quantification examining NAT10 and fibrotic markers (FN, Collagen I, and Collagen III) in BUMPT cells co-transfected with HY107856 and/or NAT10 overexpression plasmid, followed by TGF-β1 treatment (5 ng/mL, 24 h).(P-Q) Immunoblot analysis with densitometric quantification measuring JNK, p-JNK, ERK, p-ERK, and TGF-β expression in BUMPT cells subjected to the treatments described in (N-O).Data are denoted as mean ± SD (n = 6). *p< 0.05, TGF-β with Scramble group vs. Saline with Scramble group; #*P < 0.05* TGF-β1 + HY-107856 group vs. TGF-βwith Scramble group.

**Figure 9:**
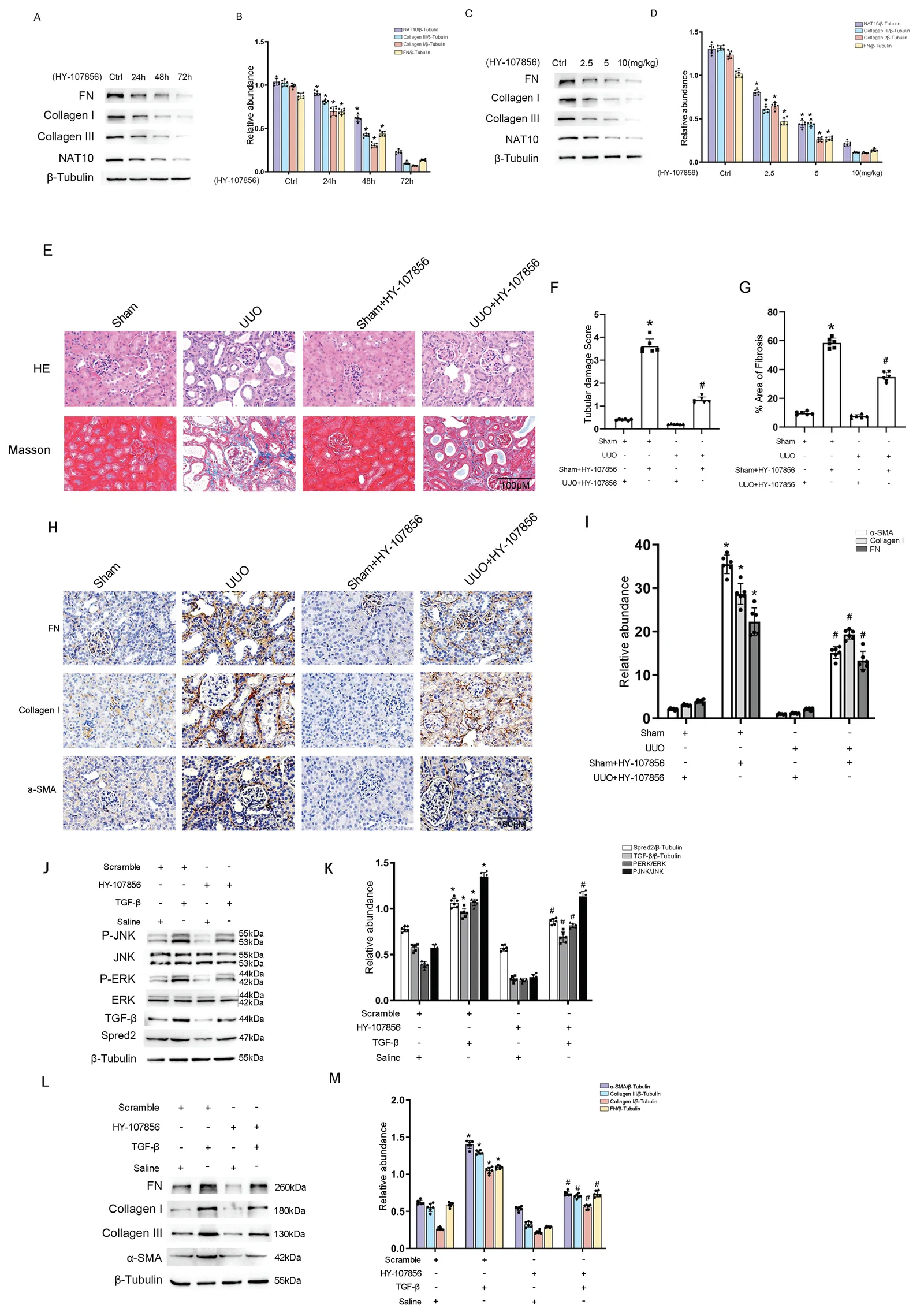
** 5-Fluorouridine ameliorates UUO-induced renal fibrosis.** (A-B) WB analysis with densitometric quantification evaluating NAT10 and fibrotic markers (FN, Collagen I, Collagen III) in kidney tissues from C57 mice harvested at 0, 12, 24, and 72 h following intraperitoneal administration of 5-Fluorouridine. (C-D) WB analysis accompanied by densitometric quantification assessing NAT10 and fibrotic markers (FN, Collagen I, Collagen III) in kidney tissues obtained from mice treated with escalating doses of 5-Fluorouridine (2.5, 5, and 10 mg/kg). (E) Representative micrographs of H&E and MTS of kidney sections from the specified groups. Scale bar = 100 μm. (F) Quantitative assessment of tubular injury scores. (G) Quantification of fibrotic areas. (H-I) Immunohistochemical staining with corresponding quantification of fibrotic markers (FN, Collagen I, and α-SMA) in kidney sections from the indicated groups. Scale bar = 100 μm. (J-M) WB analysis with densitometric quantification determining Spred2, JNK, p-JNK, ERK, p-ERK, TGF-β, and fibrotic markers (FN, Collagen I, Collagen III, α-SMA) in kidney tissues from the indicated groups, with β-Tubulin utilized as the loading control. Data are denoted as mean ± SD (n = 6). *p< 0.05, UUO group vs. sham group; #p< 0.05, UUO with HY-107856 group vs. sham with HY-107856 group.

**Figure 10:**
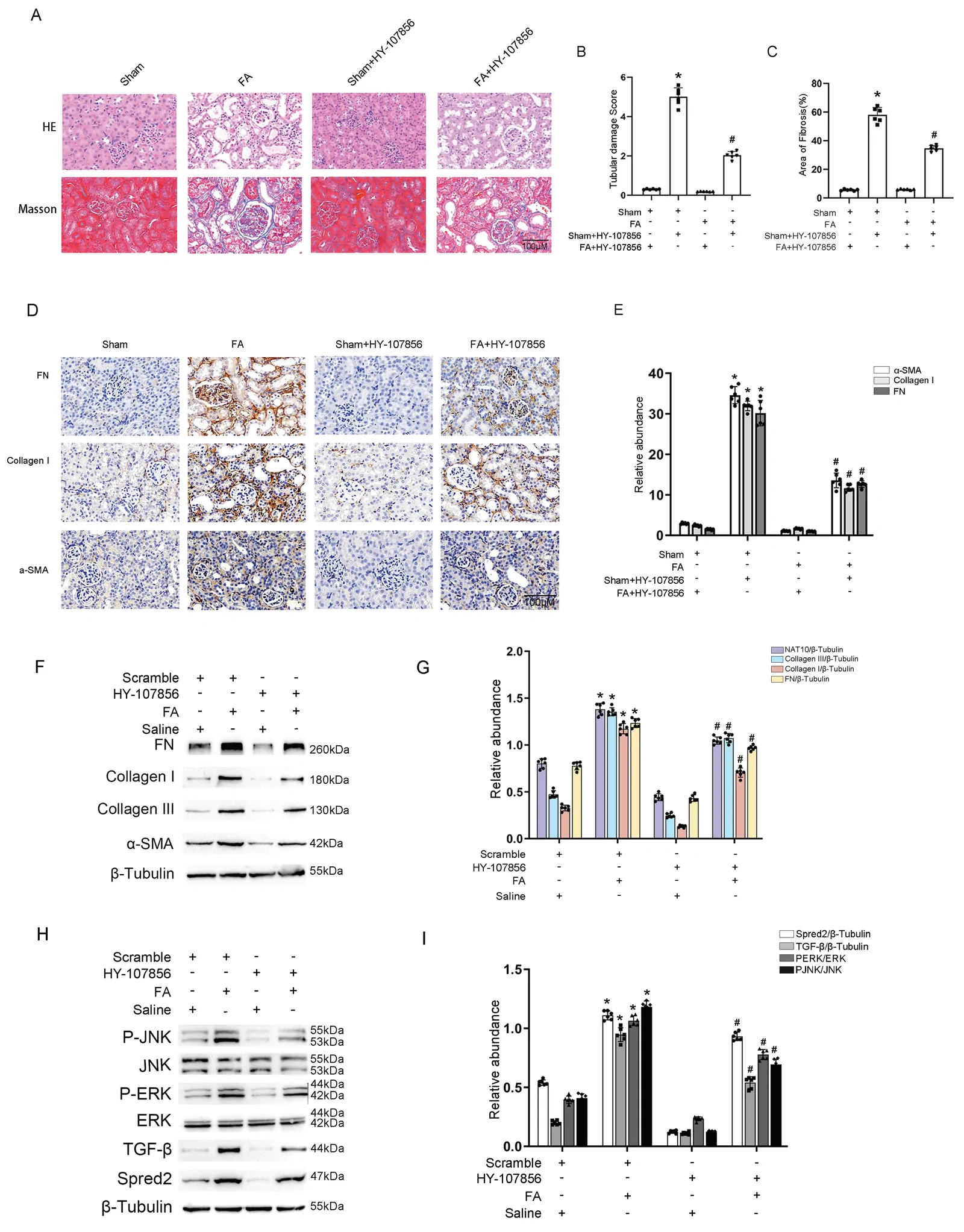
** 5-Fluorouridine ameliorates FA-induced renal fibrosis.** Mice were intraperitoneally administered FA (250 mg/kg) for five consecutive days and subsequently treated with or without 5-Fluorouridine (10 mg/kg) by every 72 hours intraperitoneal injection for 14 days. (A) Representative micrographs of H&E and MTS of kidney sections from the specified groups. Scale bar = 100 μm. (B) Quantitative evaluation of tubular injury scores. (C) Quantification of fibrotic areas. (D-E) Immunohistochemical staining with corresponding quantification of fibrotic markers (FN, Collagen I, and α-SMA) in kidney sections from the indicated groups. Scale bar = 100 μm. (F-I) WB analysis with densitometric quantification determining Spred2, JNK, p-JNK, ERK, p-ERK, TGF-β, and fibrotic markers (FN, Collagen I, Collagen III, α-SMA) in kidney tissues from the indicated groups, with β-Tubulin utilized as the loading control. Data are denoted as mean ± SD (n = 6). *p< 0.05, Folic acid group vs. sham group; #p< 0.05, Folic acid with HY-107856 group vs. sham with HY-107856 group.
